# Adsorption of copper (II) on mesoporous silica: the effect of nano-scale confinement

**DOI:** 10.1186/s12932-018-0057-4

**Published:** 2018-06-26

**Authors:** Andrew W. Knight, Austen B. Tigges, Anastasia G. Ilgen

**Affiliations:** Geochemistry Department, Sandia National Laboratories, 1515 Eubank Blvd SE, Albuquerque, NM 87123 USA

**Keywords:** Nano-scale confinement, Adsorption isotherm, Adsorption kinetics, Mesoporous silica

## Abstract

Nano-scale spatial confinement can alter chemistry at mineral–water interfaces. These nano-scale confinement effects can lead to anomalous fate and transport behavior of aqueous metal species. When a fluid resides in a nanoporous environments (pore size under 100 nm), the observed density, surface tension, and dielectric constant diverge from those measured in the bulk. To evaluate the impact of nano-scale confinement on the adsorption of copper (Cu^2+^), we performed batch adsorption studies using mesoporous silica. Mesoporous silica with the narrow distribution of pore diameters (SBA-15; 8, 6, and 4 nm pore diameters) was chosen since the silanol functional groups are typical to surface environments. Batch adsorption isotherms were fit with adsorption models (Langmuir, Freundlich, and Dubinin–Radushkevich) and adsorption kinetic data were fit to a pseudo-first-order reaction model. We found that with decreasing pore size, the maximum surface area-normalized uptake of Cu^2+^ increased. The pseudo-first-order kinetic model demonstrates that the adsorption is faster as the pore size decreases from 8 to 4 nm. We attribute these effects to the deviations in fundamental water properties as pore diameter decreases. In particular, these effects are most notable in SBA-15 with a 4-nm pore where the changes in water properties may be responsible for the enhanced Cu mobility, and therefore, faster Cu adsorption kinetics.

**Electronic supplementary material:**

The online version of this article (10.1186/s12932-018-0057-4) contains supplementary material, which is available to authorized users.

## Introduction

Developing a better understanding of the chemistry at mineral surfaces is critical to predict the fate and transport of chemical species in soils, sediments, and in porous rocks [1]. Interfacial processes, taking place at the atomic scale: ~ 10^−10^ m can manifest all the way to the scale of planet Earth: ~ 10^7^ m, and the chemistry at surfaces depend on the spatial environment of the surface [2]. Nano-scale confined domains are ubiquitous in the environment and exist in tight rocks (i.e. shale), where large fraction of pores are in the nano-scale range [2, 3]. These domains have been recognized as an important factor in the selective permeability of tight rocks, a phenomenon largely attributed to the compressed electrical double layer at the water–solid interface as the pores decrease in size [2]. These nano-scale domains can be defined as microporous (< 2 nm pore diameter), mesoporous (2–50 nm pore diameter), and macroporous (> 50 nm pore diameter) [4, 5]. When fluids reside in these nano-pores, the observed thermodynamic properties of these systems deviate from those measured in bulk non-confined phases [6]. Previous research has shown, that with decreasing pore size, the fundamental properties of water change, which is expected to affect the adsorption of ions at mineral–water interfaces. Examples of these deviations in water properties include a decrease in the dielectric constant (as much as a 50% decrease with a 1.2 nm pore) [7, 8], density, and surface tension when the pore size is less than 5 nm [9]. These deviations are largely attributed to the high ratio of mineral-surface-bound (structured) water in nano-scale confined systems relative to bulk water [9].

Stimulated by the advances in the nanotechnology industry, nanogeochemistry has emerged as an active field of research and has led to numerous observations of size-dependent phenomena, including metal adsorption on mineral surfaces [2]. Propagated from changes in water properties, an observed decrease in the solvation energies and an increase in the formation of inner-sphere complexes of metal cations can lead to enhanced adsorption [2, 10, 11]. Research has demonstrated these effects, showing an enhanced adsorption of arsenic (As) on ordered mesoporous alumina compared to non-porous activated alumina [12], and enhanced copper (Cu) and uranium (U) adsorption with decreasing hematite particle size [13, 14]. Furthermore, the adsorption mode for various adsorbed metals on zeolites with nanopores changed from outer sphere to inner sphere as the pore became smaller [15–17]. The adsorption of Cu on zeolites with narrow pores (0.26–0.74 nm) has been investigated with electron paramagnetic resonance spectroscopy and demonstrated an enhanced adsorption and increased inner sphere coordination in the zeolites with the smallest pores [15]. While these studies have illustrated the deviation in thermodynamic properties when mineral particles are limited to nano-scale, using natural mineral surfaces (i.e. hematite) or compared porous versus non-porous material, there has yet to be a study that takes a systematic approach to evaluate the adsorption properties of metal cations as a function of discrete incremental changes in the pore size.

In this study, the adsorption of Cu^2+^ was evaluated on mesoporous silica. Copper was chosen as an analyte because it is an important naturally-occurring trace element with abundance in the Earth’s crust of 50 mg kg^−1^ [18]. Copper is mined for its wide use in industrial applications, and as a result, can reach toxic concentrations in environmental systems [18–22]. At trace concentrations, Cu is essential for life, and is involved in many enzymatic reactions in mammals [18]. Industrially, Cu has applications in the electrical industry, in the production of alloys, as a catalysis, and much more [18, 19]. As a result, the total anthropogenic discharge ranges from 35,000 to 90,000 metric tons per year [18]. Therefore, Cu is a common environmental pollutant where most of the Cu release is resulting from acid mine drainage and landfill leachates [19]. Understanding the adsorption properties of Cu and how the adsorption properties are impacted by nano-scale confinement is important in broadening our understanding of the fate and transport of Cu in a variety of complex environmental and geological systems. Additionally, using Cu^2+^ to probe the reactivity of confined interfaces, allowed us to quantify size-dependent behavior. Previous research has investigated the adsorption of Cu^2+^ on functionalized ordered mesoporous silica and alumina to assess Cu^2+^ removal for water treatment purposes [21–23]. Adsorption was significantly enhanced by the addition of functional groups *N*-[3-(trimethoxysilyl)propyl]-ethylenediamine, from 0.036 mmol Cu g^−1^ on unmodified silica to 0.261 mmol Cu g^−1^ on functionalized silica [22]. Modifying the silica substrate allowed for significant removal of Cu^2+^ from aqueous solution, but in doing so, the pore geometry, volume, and reactivity were altered; and no longer represented an environmentally equivalent (e.g. silicate) system. To evaluate the effects of nano-scale confinement with environmentally relevant reactive surfaces, we focused on unmodified mesoporous silica to present the first study, to our knowledge, that directly investigated the impact of pore size on the Cu^2+^ adsorption kinetics and uptake.

Ordered mesoporous silica is an ideal candidate to assess the impacts of nano-scale confinement on the adsorption of Cu^2+^. These materials are synthetically produced via a surfactant template, creating chemically stable high surface area structures with uniform pores and a narrow distribution of pore diameters [23]. These synthetic mesoporous materials are representative of the reactive surface sites typical on mineral surfaces, containing silanol functional groups (i.e. silicates) [24]. For our investigations, we utilized three analogues mesoporous silica substrates (SBA-15) with different size pore diameters, 8 nm (SBA-15-8), 6 nm (SBA-15-6), and 4 nm (SBA-15-4) diameter, all having cylindrical pore geometry. These SBA-15 siliceous materials are produced by templating triblock copolymers to create a two-dimensional hexagonal array of non-intersecting cylindrical pores [25, 26]. This range of pore diameters (8, 6, and 4 nm) represents the scale at which nano-scale confinement effects have been determined to be most significant [8, 9]. We hypothesized that the adsorption of Cu^2+^ on mesoporous silica is pore size dependent, and suspect, as pore size decrease and confined water properties emerge, Cu^2+^ adsorption will be enhanced.

The goal of this study was to systematically assess the surface complexation of Cu^2+^ under nano-scale confinement in model mesoporous materials using batch adsorption isotherms and kinetics studies. The adsorption isotherms were fit to adsorption isotherm models (Langmuir, Freundlich, and Dubinin–Radushkevich) to extract adsorption parameters, and pseudo-first-order kinetics model to extract rate constants. This data was used to evaluate the impact of nano-scale confinement on the surface area normalized adsorptive behavior of Cu^2+^ on mesoporous silica.

## Experimental section

### General

All reagents used for batch adsorption studies were reagent grade or higher, including copper(II) nitrate trihydrate (Cu(NO_3_)_2_·3H_2_O), ammonium nitrate (NH_4_NO_3_), nitric acid (HNO_3_), and ammonium hydroxide (NH_4_OH) (Sigma-Aldrich, St. Louis, MO). Ultrapure HNO_3_ (ULTREX II, J. T. Baker, Thermo Fisher Scientific, Waltham, MA) was used for dilutions and sample preservation. Aqueous concentrations of Cu^2+^ were quantified via inductively coupled plasma mass spectrometer (ICP-MS) (NexION 350D, Perkin Elmer, Waltham, MA). All gasses and cryogenic liquids used for ICP-MS analysis and Brunauer–Emmett–Teller (BET) surface area analysis were ultrapure quality grade (Matheson, Basking Ridge, NJ) including liquid and gaseous argon (Ar) and nitrogen (N_2_), along with helium (He) gas. All mesoporous materials were purchased from Sigma Aldrich (Sigma Aldrich, St. Louis, MO). These materials included SBA-15-8, SBA-15-6, SBA-16-4 correspond to hexagonally ordered cylindrical pores with diameters of 4, 6, and 8 nm. Milli-Q H_2_O (Barnstead NANOpure Diamond, resistivity of 18.2 MΩ*cm, 0.2 μm filtered and UV irradiated) was used in the preparation of all solutions and suspensions.

### BET surface area and pore size determination

The BET surface area for each mesoporous material was obtained using two different BET surface area analyzers, an ASAP 2020 (Micrometrics, Norcross, GA) and a TriStar (Micrometrics, Norcross, GA). The procedure on both instruments was the same. Approximately 200 mg of dry mesoporous material was transferred into a tarred BET tube equipped with an airtight cap. The samples were degassed at 300 °C for 4 h and backfilled with inert He gas. Following the sample degas, the mass of the sample was updated to account for mass changes in the resulting from the degas. Next, the liquid N_2_ Dewar was filled, a thermal jacket was placed on the BET tube, the tube was placed in the sample holder, and the analysis was started. The surface area analyzer determined the BET surface area and the non-local density functional theory (NLDFT) method was used to determine pore diameter and volume for each SBA-15 substrate [26, 27].

### Thermogravimetric analysis

Thermogravimetric analysis (TGA; Thermal Analysis, SDT Q600, New Castle, DE) was performed on SBA-15-4, SBA-15-6, and SBA-15-8 to estimate the hydroxyl group density. Prior to analysis each silica material was thoroughly rinsed and dried. Briefly, approximately 600 mg of mesoporous material was added to centrifuge bottle with 200 mL of Milli-Q water and mixed on a shaker table (Orbital-Genie, Scientific Industries, INC, Bohemia, New York). Following shaking for 24 h the materials were filtered (45 μm, Pall Corporation, Ann Arbor, MI), and rinsed with Milli-Q water and suspended in 200 mL of Milli-Q water. This process was repeated two additional times. Following the final rinse, the mesoporous materials were transferred to a scintillation vial and placed in the oven (45 °C) for at least 48 h. Once dry, roughly 10 mg silica was transferred to a tarred TGA crucible (100 μL, Robocasting, Albuquerque, NM) and placed in the TGA furnace. The flow rate of Ar was 100 mL/min with an experimental sequence: (i) 20 min room temperature isothermal to equilibrate, (ii) ramp temperature at 10 °C/min from room temperature to 1000 °C.

### Batch adsorption and adsorption isotherms

Batch adsorption isotherm experiments were performed to evaluate the adsorption of Cu^2+^ on SBA-15-8, SBA-15-6, and SBA-15-4. The SBA-15 materials were rinsed prior to use, as described above (same method as TGA). Following being rinsed and dried, approximately 20 mg was weighed out and transferred to a 50 mL centrifuge tube with 5 mL of 10 mM NH_4_NO_3_ and rehydrated for at least 24 h. The electrolyte, NH_4_NO_3_, was chosen as the background electrolyte to prevent adding any metal cationic species to compete for surface adsorption sites. Following 24 h, Cu(NO_3_)_2_ was added to each 50 mL centrifuge tube with a concentration of Cu^2+^ ranging from 5 to 300 μM from a stock Cu(NO_3_)_2_ solution and brought up to 10 mL total volume with Milli-Q water. The pH was adjusted using dilute solutions of HNO_3_ and NH_4_OH to achieve a final pH of 6.0 ± 0.1 and final ionic strength of 10 ± 1 mM. Under these experimental conditions the dominant aqueous Cu species is Cu^2+^ (roughly 96%, estimated using Visual MINTEQ 3.1) [28]. A detailed Cu speciation diagram is provided in Additional file 1: Figure S1. The contents were mixed via shaker table for 24 h. After mixing, the samples were centrifuged (Allegra 25R, Beckman Coulter, Indianapolis, IN) at 3000 rpm for 10 min to separate the solid mesoporous silica from the supernatant electrolyte solution. To fully separate the phases and remove any suspended mesoporous silica, the supernatant was transferred to a 10 mL syringe fit with a 0.20 μm nylon filter (Millex-GN, EMD Millipore, Billerica, MA) and the contents pushed through to a 15 mL centrifuge tube. Ultra pure 6 M HNO_3_ was added to each sample (10 μL mL^−1^) for sample preservation to keep Cu^2+^ cations from hydrolyzing and precipitating.

The samples were diluted to a concentration range suitable for Cu quantification via ICP-MS (10–2000 ppb). Dilutions were performed using Ultrapure 2% HNO_3_ by a factor of 2, 5, 10, or 50 and kept in the refrigerator until the ICP-MS analysis. The experimental data was fit to three adsorption isotherm models: Langmuir, Freundlich, and Dubinin–Radushkevich.

#### Langmuir adsorption isotherm

The Langmuir adsorption model represents homogenous adsorption behavior in which the adsorbate occurs at definite sites and does not exceed monolayer coverage [29, 30]. In this model, adsorption occurs when an adsorbate molecule overcomes a constant adsorption activation energy, and assumes a finite number of surface sites that are all equally probable for adsorption [29, 31, 32]. The Langmuir adsorption equation is shown in Eq. 1 [29, 33],

1 $$q_{e} = \frac{{K_{L} q_{m - L} \left[ {Cu} \right]_{eq} }}{{1 + K_{L} \left[ {Cu} \right]_{eq} }}$$

where K_L_ is the Langmuir constant (L/μmol), and q_m−L_ is the surface area normalized maximum adsorption of Cu (μmol/m^2^). The experimental data was fit to the linear form of the Langmuir adsorption isotherm is shown in Eq. 2.

2 $$\frac{1}{{q_{e} }} = \frac{1}{{q_{m - L} }} + \frac{1}{{K_{L} q_{m} \left[ {Cu} \right]_{eq} }}$$

From the Langmuir adsorption model we estimated the dimensionless equilibrium parameter (R_L_) shown in Eq. 3.

3 $$R_{L} = \frac{1}{{1 + K_{L} \left[ {Cu} \right]_{inital} }}$$

The equilibrium parameter provides an understanding of the favorability of the adsorption as a function of the initial concentration of adsorbate, when R_L_ > 1 the adsorption is unfavorable, 0 < R_L_ < 1 the adsorption is favorable, and when R = 0 the adsorption is irreversible [30, 34].

#### Freundlich adsorption isotherm

In contrast to the Langmuir adsorption model, in which surface does not exceed monolayer coverage of the adsorbate, the Freundlich adsorption model describes heterogeneous adsorption behavior in which the adsorption coverage can exceed monolayer coverage [35]. The Freundlich adsorption equation is shown in Eq. 4,

4 $$q_{e} = K_{F} \left[ {Cu} \right]_{eq }^{{\frac{1}{n}}}$$

where K_F_ is related to the surface area normalized maximum adsorption of Cu (μmol/m^2^) and n is dimensionless term that indicates the extent of surface heterogeneity. The linear form of the Freundlich adsorption model is shown in Eq. 5.

5 $$\log q_{e} = \log K_{F} + \frac{1}{n}\log \left[ {Cu} \right]_{eq}$$

It should be noted that the Freundlich isotherm does not accurately determine the absolute maximum adsorbed Cu [36]. This is due to the fact that q_e_ is directly proportional to [Cu^2+^]_eq_, and therefore q_e_ will increase as [Cu^2+^]_eq_ increases. Yet, the K_F_ value can be used as a qualitative comparison to assess the relative adsorption maximum values of Cu on mesoporous silica surfaces.

#### Dubinin–Radushkevich adsorption isotherm

The final adsorption isotherm considered to evaluate the adsorption of Cu on mesoporous materials was the Dubinin–Radushkevich adsorption isotherm. This empirical adsorption model was first used to describe a pore filling mechanism and is generally used to express adsorption processes that occur via homogeneous and heterogeneous processes [37]. Equation 6 is the Dubinin–Radushkevich adsorption model,

6 $$q_{e} = q_{m - DR} *e^{{ - k_{DR} \varepsilon^{2} }}$$

where q_m−DR_ is the maximum surface area normalized amount of Cu adsorbed on the mesoporous surfaces (μmol/m^2^), and k_DR_ is proportional to the adsorption energy (mol^2^/kJ^2^). The Polanyi potential, ε, is defined by Eq. 7 [38],

7 $$\varepsilon = RT*\ln \left( {1 + \frac{1}{{\left[ {Cu} \right]_{eq} }}} \right)$$

where R is the gas constant (kJ/K*mol) and T is the temperature (K). The linear form of the Dubinin–Radushkevich adsorption model is shown by Eq. 8.

8 $$\ln q_{e} = \ln q_{m - DR} - k_{DR} \varepsilon^{2}$$

The fitting parameters q_m−DR_ and k_DR_ are determined by the intercept and slope, respectively, of a the linear line when plotted ln (q_e_) versus ε^2^.

### Kinetic studies

Kinetic studies were performed to determine the rate law of the Cu adsorption on mesoporous materials, and to quantify the effect of nano-scale confinement on the adsorption rates. For these studies, 100 mg of hydrated mesoporous silica was transferred to a 100 mL beaker with 25 mL of 10 mM NH_4_NO_3_, a stir bar set to vigorously mix the solution at 300 rpm and enough DI water to achieve a final volume of 50 mL. The solution pH was adjusted using HNO_3_ and NH_4_OH to a pH = 6.0 ± 0.1. Following the pH adjustment, a known amount of Cu^2+^ was spiked into the solution (5–10 μM Cu). The time and pH were recorded at this point and represented time = 0. The pH was continuously monitored by the pH probe as 3 mL aliquots were removed at set time points during the experiment and transferred to a 10 mL syringe and filtered into a 15 mL centrifuge tube containing 30 μL ultra pure 6 M HNO_3_. The kinetic studies ran for 6 h to establish an equilibrium value for adsorbed Cu (q_e_). This procedure was repeated at three concentrations of Cu^2+^ for each silica material.

Following the kinetic experiments, the samples representing unique time points (minutes) were diluted by a factor of 3 with Ultrapure 2% HNO_3_ for Cu quantification by ICP-MS. The diluted samples were kept in the refrigerator until sample analysis for Cu.

#### Pseudo-first-order adsorption kinetics

Batch kinetic adsorption studies were performed and fit to a pseudo-first-order reaction model. The rate constants for the adsorption of Cu on SBA-15-4, SBA-15-6, and SBA-15-8 were determined from the model fit. The pseudo-first-order reactions occur when the reaction is second order, however, one of the reactants is in excess and largely remains unchanged [39]. In this case, three different dilute initial Cu^2+^ concentrations were reacted with 100 mg of mesoporous material. This is consistent with the fact that we had low Cu^2+^ concentrations and high silica reactive surface area.

The rate law representing a pseudo-first-order reaction is shown in Eq. 9.

9 $$Rate = k*\left[ {Reactive Surface} \right]\left[ {Cu} \right]$$

Since the concentration of the reactive surface is in excess and essentially remains unchanged throughout the reaction coordinate, the rate equation can be reduced to Eq. 10:

10 $$Rate = k^{\prime}\left[ {Cu} \right]$$

where k′ is related to k and the reactive surface concertation [39]. The integrated pseudo-first-order rate equation is shown in Eq. 11,

11 $$\ln \left( {q_{e} - q_{t} } \right) = \ln q_{e} - kt$$

where, q_e_ is the equilibrium adsorbed Cu, *q*_*t*_ is the adsorbed Cu at time = *t*, *k* is the pseudo-first-order rate constant (min^−1^), and *t* is time (min).

### Copper analysis

The Cu content in each sample was quantified by ICP-MS as described previously [40]. Briefly, standards were prepared with concentrations ranging from 10 to 2000 ppb and used to generate a calibration curve to determine concentration of Cu in each sample from intensity. The stock solutions and reference solutions of identical experimental matrix (in the absence of SBA-15) were analyzed with the samples. The surface area normalized amount of adsorbed Cu (q), in μmol/m^2^, was determined from Eq. 12:

12 $$q = \frac{{\left( {\left[ {Cu} \right]_{i} - \left[ {Cu} \right]_{eq} } \right)*v}}{{\left( {m*A} \right)}}$$

where [Cu]_i_ and [Cu]_eq_ are the initial and equilibrium concentrations (measured by ICP-MS) of Cu in μM, *v* is the volume in L, *m* is the mass of mesoporous material in g, and *A* is the surface area of that mesoporous materials as determined by BET surface area analyzer in m^2^/g. The q values were then used to determine adsorption isotherm fits and kinetic rates of Cu adsorption on SBA-15-8, SBA-15-6, and SBA-15-4 substrates.

## Results and discussion

From studies investigating Cu adsorption on SBA-15-8, SBA-15-6, and SBA-15-4, we systemically evaluated the effects of nano-scale confinement. The sum of these effects provide insight into how nano-scale confinement can enhance metal adsorption in meso- and nano-porous materials.

### BET surface area and pore size distribution

The BET surface areas, pore diameters, pore volumes, and percent pore surface area were determined for each SBA-15 used in this study. The results are summarized in Table 1. Surface area and pore size distributions were evaluated on two different BET analyzers as the experimental surface area trend did not agree with the surface areas provided by the supplier. However, the average pore diameter trend did agree with the supplier reported value. We observed a decrease in the total BET surface areas of 661 ± 5 m^2^/g, 603 ± 16, and 582 ± 13 m^2^/g as the pore size decreased from 7.0 ± 0.3, 5.2 ± 0.2, 4.4 ± 0.1 nm for SBA-15-8, SBA-15-6, and SBA-15-4, respectively. Furthermore, a recent study demonstrated a similar trend with synthesized SBA-15 substrate, as the pore diameter decreased, the BET surface area decreased as well [25]. The experimental values of the BET surfaces areas reported in Table 1 were used to normalize the adsorption of Cu to the total surface area of the substrate.

**Table 1:** Pore size and BET surface area of SBA-15-8, SBA-15-6, and SBA-15-4

Material	Pore size (nm)	BET surface area (m^2^/g)	Pore volume (cm^3^/g)	% surface area from pores	–OH-density (–OH/nm^2^)
SBA-15-8	7.0 ± 0.3	661 ± 5	1.21 ± 0.03	89 ± 6	1.8 ± 0.2
SBA-15-6	5.2 ± 0.2	603 ± 16	0.87 ± 0.03	80 ± 5	1.9 ± 0.2
SBA-15-4	4.4 ± 0.1	580 ± 13	0.67 ± 0.04	75 ± 6	2.3 ± 0.2

The BET surface areas were used to calculate the surface area normalized Cu adsorption

The pore diameters were determined using the NLDFT method [26, 27]. The pore size distribution plots are shown in Additional file 1: Figure S2. The mesopore diameter is relatively narrow distribution resulting in average pore diameters and volumes reported in Table 1. As reported in previous pore size distributions of SBA-15 materials, there are micropores present as well [26, 41, 42]. The mesopores exist in the main channels as templated from the removal of the calcined surfactant, whereas the micropores co-exist due to intrawall pores that develop as a consequence of the removal of the surfactant template creating structural irregularities [43]. This can be seen in the SBA-15-4 plot (Additional file 1: Figure S2), in which the pore size distribution is bimodal with peaks centered around 4.4 and 2 nm. In contrast, the SBA-15-8 (Additional file 1: Figure S2) shows a primary contribution from the mesopores centered around 8 nm, and a small contribution of micropores centered around 2 nm. For this study, we considered the average pore size diameter to assess the effects of nano-scale confinement. While the observed effects may be attributed to the increased presence of micropores, the micropores are defined by one data point and we believe appropriate nano-scale confinement conclusions can be drawn from adsorption deviations of Cu with these substrates.

### Thermogravimetric analysis

**Fig. 1:**
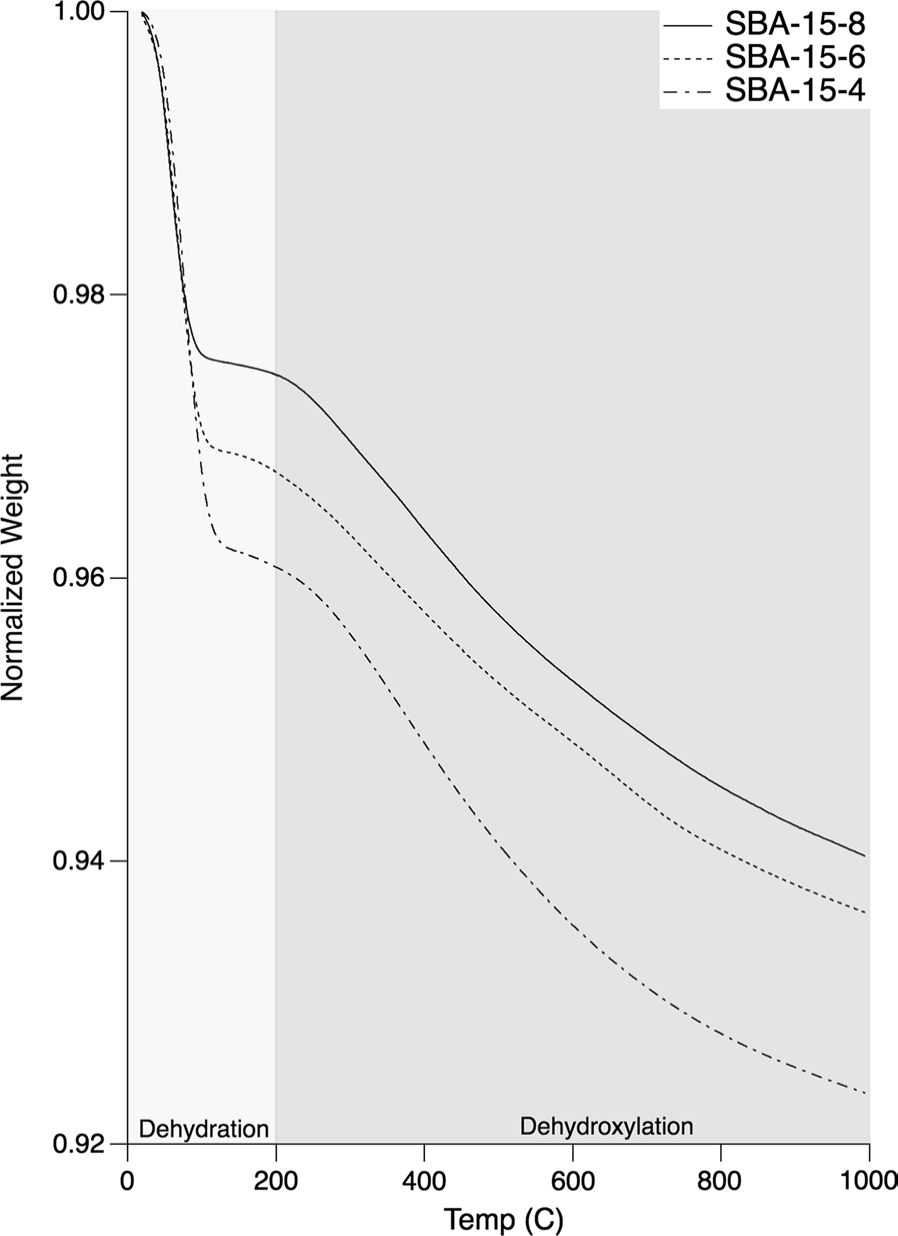
Thermogravimetric Analysis of SBA-15-4, SBA-15-6, and SBA-15-8. This analysis was run in argon (100 mL/min), with an isothermal equilibrium for 20 min before ramping up to 1000 °C at a rate of 10 °C/min

The results of the TGA analysis are shown in Fig. 1. This plot shows the percent mass loss of SBA-15-8, SBA-15-6, and SBA-15-4 as a function of temperature. From the TGA plots the surface –OH group density was estimated and reported in Table 1. The first observable mass loss feature in Fig. 1 between 20 and 200 °C was attributed to presence of physisorbed water [44]. Following the water loss, the mass loss from 200 to 1000 °C was primarily attributed to surface dehydroxylation [44]. There are likely to be internal –OH groups, however, these groups are not typically dehydroxylated until > 900 °C [44]. As a result, the average –OH surface densities (–OH/nm^2^) were determined to be 1.8 ± 0.2, 1.9 ± 0.2, 2.1 ± 0.2 for SBA-15-8, SBA-15-6, and SBA-15-4, respectively. These –OH surface densities strongly agree with previously reported accessible –OH densities of SBA-15 of 1.7 –OH/nm^2^ [45, 46]. The –OH density also corresponds with the observed wettability, as seen by the magnitude of desorption of physisorbed water. An increase in the –OH density would lead to a more hydrophilic surface. The –OH group density appears to inversely correlate with the pore size.

### Cu adsorption isotherms and nano-scale confinement effects

**Fig. 2:**
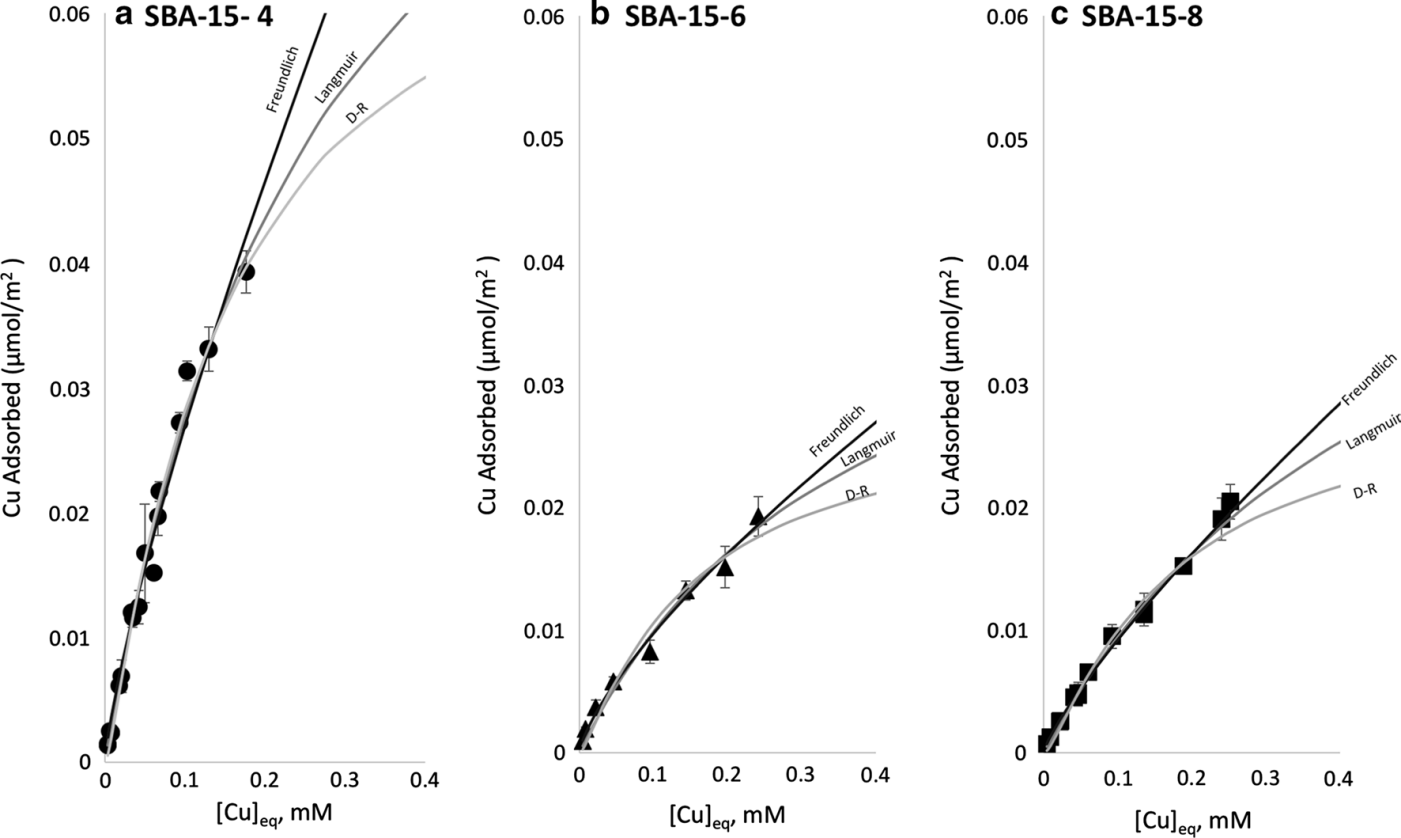
Adsorption isotherm plots showing the adsorption of Cu on mesoporous materials. **a** Cu adsorption on SBA-15-4. **b** Cu adsorption on SBA-15-6 and **c** Cu adsorption on SBA-15-8 fit with Langmuir, Freundlich and Dubinin–Radushkevich isotherm models

The adsorption of Cu on SBA-15-8, SBA-15-6, and SBA-15-4 are shown in Fig. 2, where the amount of Cu (μmol m^−2^) adsorbed was plotted versus the equilibrium concentration of Cu^2+^. The data was fit to adsorption isotherm models: Langmuir [29], Freundlich [35], and Dubinin–Radushkevich [37]. Copper adsorption onto the surface of SBA-15 was poor overall, with the maximum measured surface loading of Cu of 0.020 ± 0.001, 0.019 ± 0.002, and 0.039 ± 0.002 μmol m^−2^ of substrate for SBA-15-8, SBA-15-6, and SBA-15-4, respectively, when [Cu]_initial_ = 300 μM. The maximum measured surface loading of Cu was equal within experimental error for SBA-15-8 and SBA-15-6, however for SBA-15-4, the maximum measured surface loading was nearly double. While we did observe differences in the surface –OH group densities (1.8 vs 2.1 –OH/nm^2^ for SBA-15-8 and SBA-15-4), we believe those differences are not sufficient to explain the observed differences between SBA-15-4 over SBA-15-6 and SBA-15-8. To add, the available reactive surface –OH sites remained in 100-fold excess to the maximum Cu values all cases. Because the number of –OH groups remains in 100-fold excess over adsorbed Cu atoms, we believe that the observed differences in adsorption should be attributed to the effects of nano-scale confinement. Likewise, the estimated percent of occupied –OH surface sites (assuming mono-dentate inner-sphere Cu adsorption complex [19]) never exceed 1%. These calculations may suggest that Cu requires a specific surface conformation of silanol groups to bind [46]. This hypothesis will be investigated by X-ray adsorption spectroscopic studies.

**Fig. 3:**
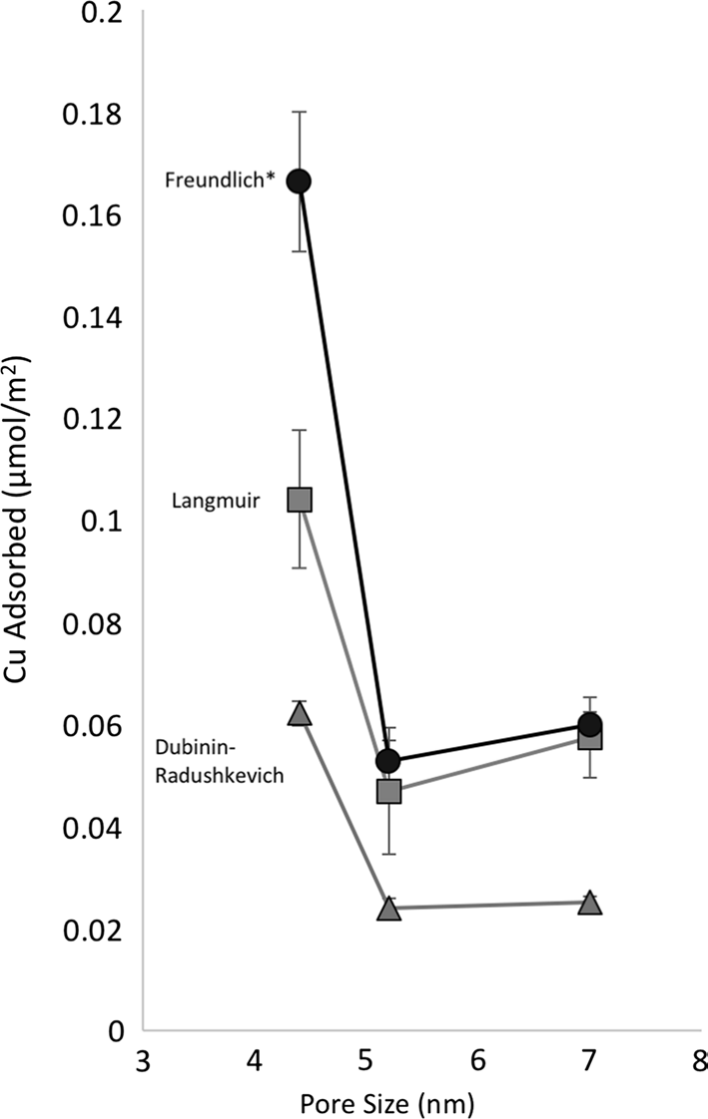
Surface area normalized adsorption maximum values of Cu calculated from Langmuir, Freundlich, and Dubinin–Radushkevich isotherm models as a function of pore size. *Freundlich adsorption model does not provide an accurate absolute maximum adsorption value; however, can be used to assess relatively adsorption maximums

The adsorption of Cu was fit to Langmuir, Freundlich, and Dubinin–Radushkevich isotherms using IGOR Pro 7 curve fitting using equations representing each model. For these studies pH = 6 was chosen because Cu^2+^ is the dominant aqueous species (Additional file 1: Figure S1, [Cu] = 0.1 mM), as well as our target initial concentrations of Cu^2+^ were below the solubility limit; however, at [Cu]_initial_ > 0.3 mM, Cu began to precipitate. Further, pH = 6 was chosen to maximize the adsorption of Cu, based on the previous adsorption studies which illustrated that Cu was poorly adsorbed onto amorphous silica at pH < 6 [19, 20]. For these reasons; (i) limited Cu solubility, (ii) relatively poor Cu adsorption, all three adsorption isotherms were close to being linear (Fig. 2), and could be fit with either of the three isotherm models, as seen from r^2^ values > 96% (and most fits were > 99%) in Table 2. The observed trends were consistent among each of the adsorption models. It was observed that SBA-15-4 had the largest uptake of Cu (normalized to the surface area), followed by SBA-15-6 and SBA-15-8; which were generally within experimental error as seen in Fig. 3 and Table 2. Figure 3 presents the surface area normalized adsorption maximum values as a function of pore size for each adsorption isotherm model. It is important to note, that as a result of the range limitation of the adsorption isotherm and low adsorption, the isotherms are limited to mostly the linear region. Because of this, the uncertainties of the maximum adsorption values are relatively large. Even so, the observed differences in the surface area normalized adsorption of Cu on silica with different pore sizes illustrates nano-scale confinement effects. These effects are quantified from the terms q_m−L_, K_F_, and q_m−DR_; referring the model determined maximum adsorption value for Langmuir, Freundlich, and Dubinin–Radushkevich isotherm, respectively. While the adsorption of Cu on SBA-15-8 and SBA-15-6 were within error (for Langmuir and Dubinin–Radushkevich) or slightly different (Freundlich), the adsorption of Cu on SBA-15-4 was significantly enhanced for all isotherm models. This observation agrees with previously reported results suggesting enhanced metal cation adsorption when pore sizes or particles are less than 5 nm [2, 9].

**Table 2:** Fitting parameters obtained from the linear transformation of the data for Langmuir, Freundlich, and Dubinin–Radushkevich adsorption models

Material	Langmuir fit			Freundlich fit			Dubinin–Radushkevich fit		
Substrate	K_L_, L/µmol	q_m−L_, µmol/m^2^	r^2^	n	K_F_ (µmol/m^2^)	r^2^	K_D–R_, mol^2^/kJ^2^	q_m−DR_ µmol/m^2^	r^2^
SBA-15-8	2.0 ± 0.4	0.057 ± 0.008	0.996	1.23 ± 0.03	0.060 ± 0.002	0.998	3.4 × 10^−8^ ± 8 × 10^−9^	0.0251 ± 0.001	0.962
SBA-15-6	2.7 ± 0.6	0.05 ± 0.01	0.996	1.36 ± 0.08	0.053 ± 0.004	0.990	3.0 × 10^−8^ ± 8 × 10^−9^	0.024 ± 0.002	0.984
SBA-15-4	3.6 ± 0.6	0.10 ± 0.01	0.994	1.27 ± 0.06	0.17 ± 0.01	0.992	2.9 × 10^−8^ ± 8 × 10^−9^	0.0622 ± 0.002	0.982

An assessment of the qualitative adsorption parameters (K_L_, *n*, and K_D–R_) shows that the adsorption affinities are generally similar for all three silica materials [29, 35, 37]. The qualitative adsorption parameter K_L_ and K_D–R_ (K_D–R_ term in inversely proportional to adsorption affinity) suggest the adsorption affinity in the Langmuir and Dubinin–Radushkevich isotherm models are pore size dependent, while *n* has no pore size dependency trend as shown in Table 2. These terms qualitatively describe the adsorption affinity, suggesting there may be an increased adsorption affinity of Cu as the pore size decreases.

Additional information can be gleaned from the values of K_L_. From the K_L_ value, R_L_ can be approximated from Eq. 3. A plot of R_L_ versus [Cu]_initial_ (Additional file 1: Figure S3) estimates the reaction coordinate based upon differences in the K_L_ values. The values of R_L_ decrease with increasing Cu^2+^ concentration and remain between 1 and 0. This shows, as Cu^2+^ increases in the system, surface sites become occupied, thus the surface reaction decreases, but remains favorable. It was also found that the K_L_ for SBA-15-4 pore was the lowest, indicating the most rapid decay of the R_L_ term as a function of [Cu]_initial_. At the maximum [Cu]_initial_ = 0.3 mM, the R_L_ values are 0.63, 0.55 and 0.48 for SBA-15-8, SBA-15-6, and SBA-15-4, respectively. At the maximum adsorbed values of Cu, only 1% of the total –OH groups were occupied. The reactive surface sites are in excess compared to Cu and therefore the reaction favors the formation of surface complexes. Because these values are between 1 and 0, the surface adsorption reaction is still favorable; which suggests either, (i) the monolayer surface coverage has not reached a maximum value, or (ii) that multi-layer adsorption (i.e. dimerization, polymerization, or precipitation) occurs even at low surface coverages.

Additional work is planned to assess the coordination chemistry of Cu as a function of pore size to determine whether mechanistic changes result from nano-scale confinement. The current reaction condition, pH = 6, is above the point of zero charge, p_z_c, of SBA-15 silica (p_z_c = 4.2) [47]. As a result, the silica surface is expected to be negatively charged, and the electrostatic interactions could be driving forces leading to the adsorption of free Cu^2+^ cations onto the silica surface. Building off the X-ray absorption data analysis by Cheah et al., Cu forms inner-sphere surface complexes with amorphous silica [19, 20]. Additional analyses observed short Cu–Cu distances (2.58–2.59 Å), suggesting the formation of dimeric species adsorbed onto the amorphous silica surface. The bonding mode was concluded to be mono-dentate, demonstrated from the presence of only one unique Cu–Si distance (~ 3.0 Å), whereas a bidentate-bridging bonding mode would have resulted in two unique Cu–Si distances, distinguishable spectroscopically [19, 20]. However, with decreasing pore size, to a pore regime where nano-scale confinement effects may become apparent, it is unclear if the adsorption mode and Cu adsorption complex geometry would remain constant. Ferreira et al. demonstrated that Cu contains two electron orbitals of similar energy, therefore is subject to Jahn–Teller distortion in its octahedral coordination environment—creating a weak point in the hydration shell [15, 17]. As a consequence, Cu was shown to change its coordination environment from outer-sphere to inner-sphere when adsorbed in narrow pores of mordenite (limiting pore diameter 0.26 nm) [15, 17]. As shown by the significantly enhanced surface area normalized adsorption of Cu on SBA-15-4 over SBA-15-6 and SBA-15-8, our data may suggest changes in the adsorption complex geometry due to changes in water properties and steric strain of pore confinement. Our data agrees with previous studies showing that the changes in water properties and subsequent tendency for cations to form inner-sphere complexes preferentially enrich trace elements in nanopores [48, 49]. Wang et al. demonstrated a nearly 10-fold increase in the surface area normalized adsorption of both zinc (Zn^2+^) and arsenate (AsO_4_^3−^) on nano-porous alumina compared to alumina particles. Wang et al. attributed the observed increase in adsorption to the increase in the surface charge density inside nano-pores, and the impact of a larger proportion of structured water on ion hydration [2, 48, 50]. Furthermore, another study of uranium U^6+^ adsorption and desorption on mesoporous silica (MCM-41), demonstrated the formation of polymeric species—and eventual precipitation—inside the pores [51]. It is possible that the surface area normalized adsorption of Cu is enhanced in small mesopores (4 nm) due to Cu losing it hydration shell thus increasing its reactivity towards dimerization and surface adsorption.

### Effect of nano-scale confinement on adsorption kinetics

**Fig. 4:**
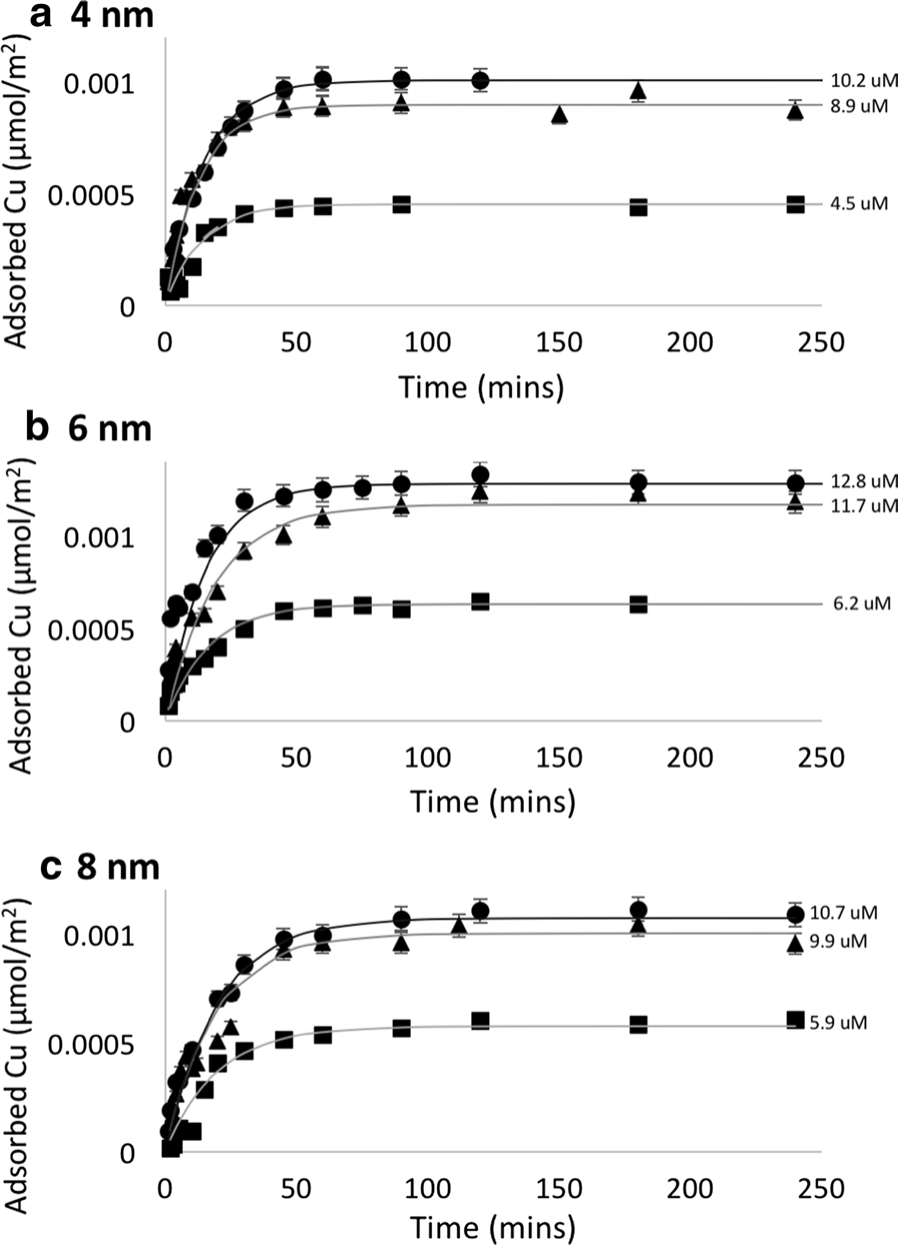
Surface area normalize adsorption of Cu versus time in minutes for **a** SBA-15-4, **b** SBA-15-6, and **c** SBA-15-8. The final equilibrium concentrations are shown for each line

**Fig. 5:**
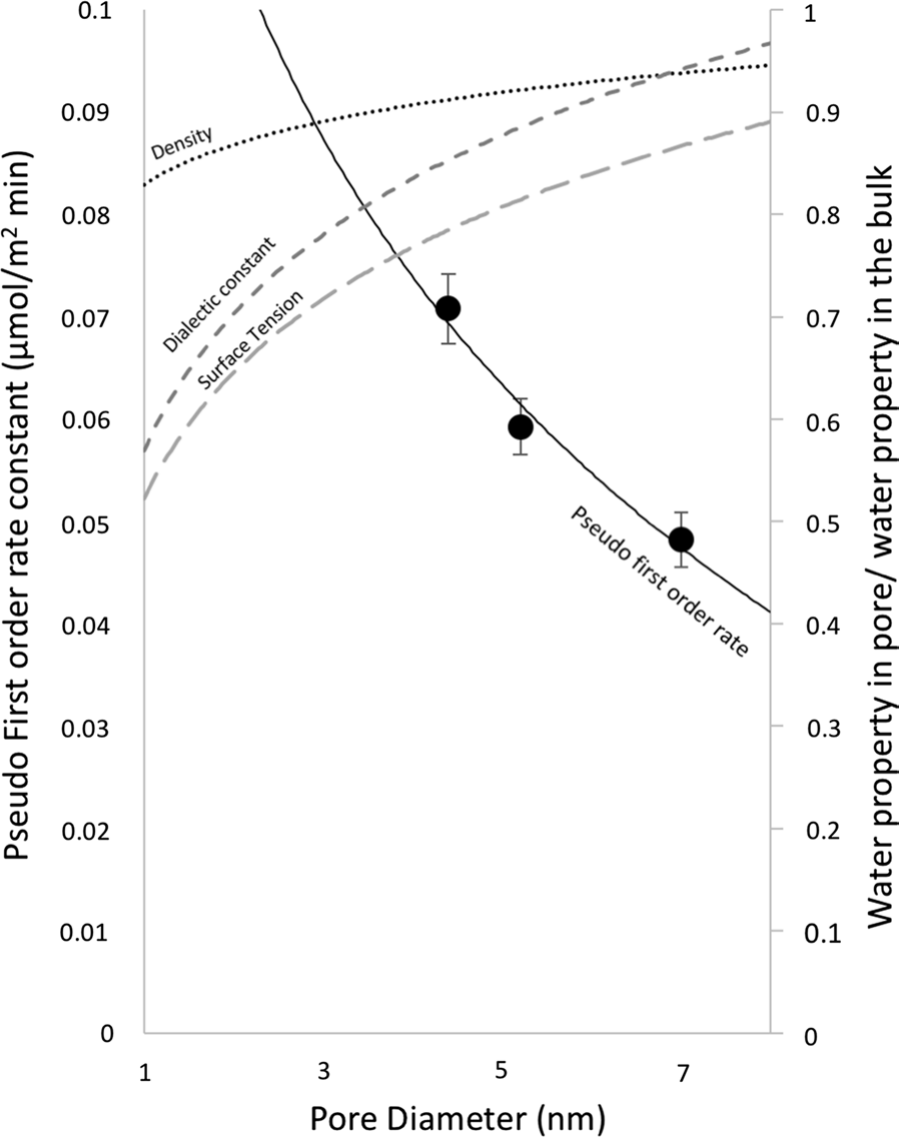
Pseudo-first-order rate constant for Cu adsorption on mesoporous silica (μmol/m^2^ min) as a function of pore size. Approximated water properties as function of pore size were obtained from previous studies for dielectric constant [8], surface tension [9], and density [9]

The surface area-normalized time dependent Cu adsorption on SBA-15-8, SBA-15-6, and SBA-15-4 fit with pseudo-first-order kinetic model is shown in Fig. 4. The adsorption rate constants were obtained from the slope of the linear regression fit to the integrated rate equation. The experimentally determined rate constants show that the adsorption of Cu on SBA-15-4 is fastest, as is summarized in Fig. 5. The rate constants representing the adsorption kinetics of Cu on mesoporous silica are 0.048 ± 0.003, 0.059 ± 0.003, and 0.071 ± 0.003 μmol/m^2^min for SBA-15-8, SBA-15-6, and SBA-15-4, respectively.

As seen in Fig. 5, the pseudo-first-order kinetic rates are inversely proportional to pore diameter; as the pore diameter decreases, the kinetic rate increases. Previous studies have summarized the dependence of surface tension [9], density [9], and dielectric constant [8] of water. Those parameters were approximated from the publications and added to Fig. 5 to compare to our data. As seen from Fig. 5, the changes in density, surface tension, and dielectric constant of water have a negative correlation with the adsorption kinetics quantified in our study. From these trends, we have concluded that the rate of Cu adsorption could be related to the decrease in the surface tension, density, and dielectric constant of water. These changes in water properties could increase the propensity of Cu to lose its hydration sphere and from inner-sphere complexes with the surface. We believe it is reasonable that these changes in the water properties will impact the rate of adsorption of Cu; as the pore decreases in size, a decrease in surface tension and density would allow more rapid flow inside the pores; a decrease in the dielectric constant would lead to a decrease in the hydration energy of the Cu atom, thus allowing for more rapid adsorption on the surface.

#### Intraparticle diffusion

Further data interpretation of the Cu adsorption kinetics was evaluated using the Weber and Morris intraparticle diffusion model [31, 33, 52, 53]. This model provides additional information regarding the modes of adsorption and breaks down the adsorption rate into external mass transfer and intraparticle diffusion steps [34]. The intraparticle model suggests that adsorption occurs via three steps:

1. External mass transfer of adsorbate from the bulk solution to the adsorbent surface;
2. Adsorbate diffusion from external surface into the adsorbent pores;
3. Adsorption of adsorbate at the surface reactive sites.

Step three is assumed to be rapid in comparison to the two diffusion steps; therefore, the overall rate is a combination of external mass transfer and intraparticle diffusion [31, 52]. The intraparticle diffusion plots were generated by plotting q_t_ versus t^0.5^, as described in Eq. 13.

13 $$q_{t} = k_{i} t^{0.5}$$

where k_i_ is the diffusion rate constant $\frac{\mu mol}{{m^{2} *\min^{0.5} }}$. The resulting plots from this model are best represented with a multi-linear regression. The multi-linearity represents the rate dependency on film diffusion early in the reaction, and the eventual rate dependence on intraparticle diffusion in later time regimes [21, 52]. For our analysis, we utilized the Piecewise Linear Regression (PLR) Excel Spreadsheet made available to us [52]. This spreadsheet performs a statistical analysis based upon F-ratio and evidence testing to determine the best fit of the intraparticle diffusion plot [52].

**Fig. 6:**
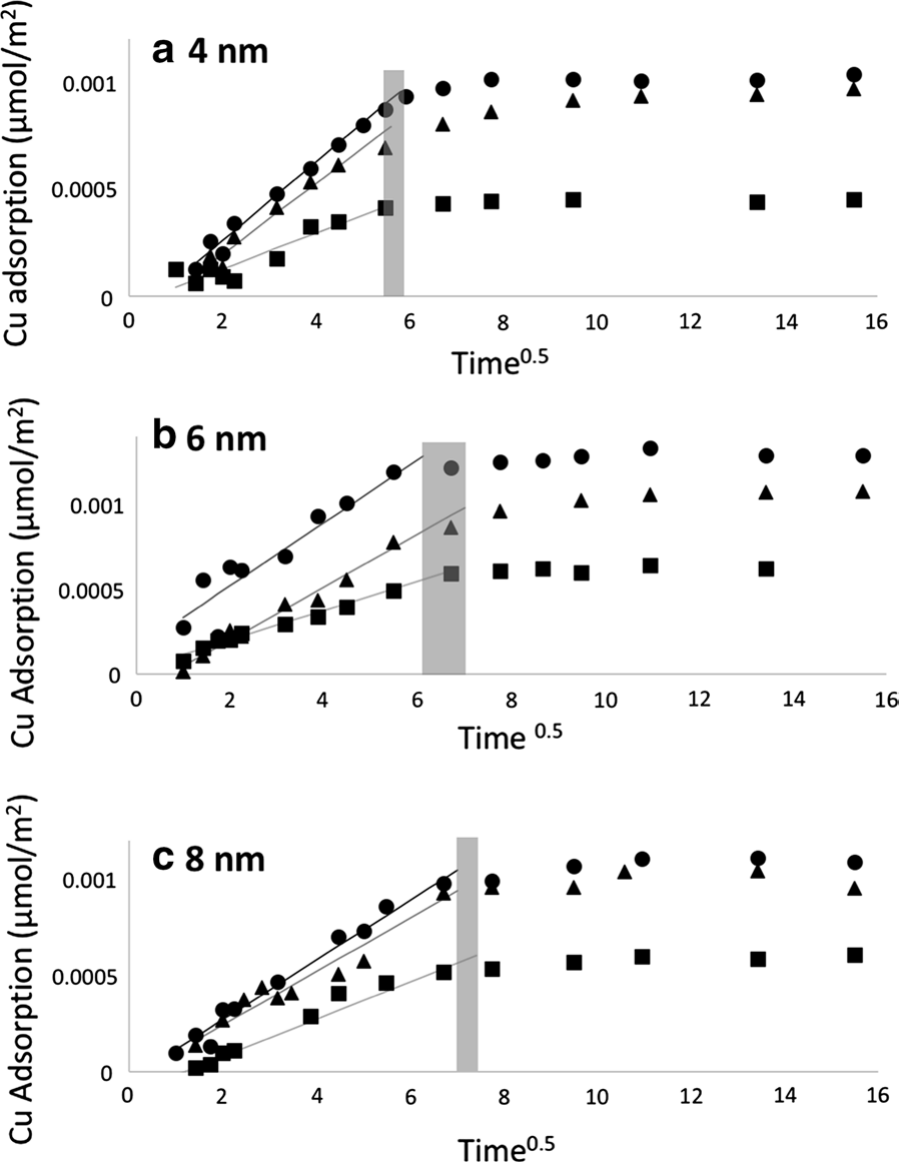
Intraparticle diffusion plots for the adsorption of Cu with on **a** SBA-15-4, **b** SBA-15-6, and **c** SBA-15-8. The section of the intraparticle diffusion model representing the external mass transfer was fit with a linear regression line based upon Piecewise Linear Regression [52]. The shaded regions represent the time break points used for the slope analysis

The intraparticle diffusion plots are shown in Fig. 6. Each intraparticle diffusion plot was fit using the PLR spreadsheet and fit with linear segments and breakpoints of those functions that best represented the system. As a general trend, our data shows adsorption begins with a period of rapid film diffusion, followed by subsequent slower adsorption step representing intraparticle diffusion, until equilibrium is reached. Each kinetic profile was fit with two linear segments and the slopes of the film diffusion section was fit to the plots in Fig. 6. The breakpoints were calculated to be 50 ± 2, 49 ± 1, and 32 ± 8 min for SBA-15-8, SBA-15-6, and SBA-15-4, respectively. These breakpoints were calculated as an average from various initial concentrations of Cu. From the breakpoints, the time regimes dependent on external mass transfer or intraparticle diffusion were established and fit with linear regression lines.

**Fig. 7:**
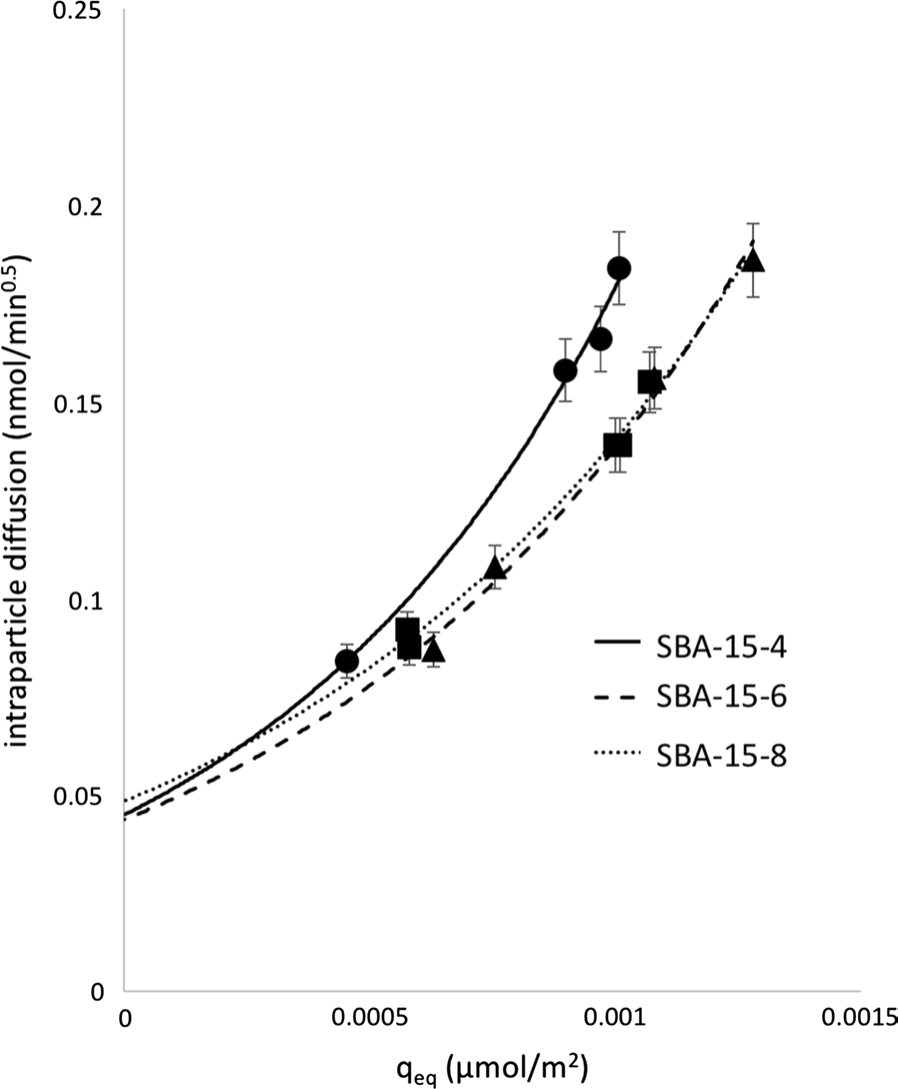
Intraparticle diffusion constant, k_i_, versus equilibrium adsorption of Cu, q_eq_, on SBA-15-4, SBA-15-6, and SBA-15-8

The slopes of linear segments, k_i_, describing the external mass transfer for SBA-15-8, SBA-15-6, and SBA-15-4 represent the diffusion rate constant. The k_i_ values versus the q_max_ is shown in Fig. 7. As the q_max_ increases, the k_i_ value increases. This suggests that with higher [Cu^2+^] in the system, the external mass transfer from the bulk to the silica film is more rapid. This observed trend is true for all three SBA-15 substrates. Additionally, the curves in Fig. 7 for SBA-15-8 and SBA-15-6 are nearly identical, however the curve representing the relationship between k_i_ and q_eq_ for SBA-15-4 is different. From this plot, it can be concluded that the film diffusion rate for Cu adsorption on SBA-15-4 is more rapid than on the larger pores. Once again, this can be attributed to the net effects of changes to the water properties (i.e. dielectric constant, density, and surface tension) and subsequent interaction of water with Cu and water with the silica surface.

## Conclusions and future work

This experimental study presents a systematic approach to quantify the effects of nano-scale confinement on metal adsorption. While nano-scale confinement effects have been observed in mesoporous systems, this is the first, to our knowledge, study quantifying nano-scale confinement effects on the adsorption of Cu on mesoporous silica. Through an analysis of the surface area normalized adsorption isotherms and adsorption kinetics of Cu on SBA-15-8, SBA-15-6, ad SBA-15-4, we concluded that nano-scale confinement enhances both the adsorption maximum, and Cu adsorption reaction rate. Evidence of this is shown in the significant increase in the surface area normalized adsorption maximum of Cu on SBA-15-4 compared to both SBA-15-6 and SBA-15-8 across all isotherm models. Further, the pseudo-first-order reaction rate constant increased with decreasing pore size. The intraparticle diffusion model was applied, and it illustrates that external mass transfer diffusion constant increases with decreasing pore size, and we postulate that this rapid film diffusion in 4 nm pores was responsible for the observed increase in reaction rate. Future work should address molecular-scale speciation of Cu associated with mesoporous silica surfaces, using spectroscopic approaches.

We believe this study will aid in the understanding of the impacts of cation adsorption in geologically relevant mesopores. This will increase our ability to anticipate cation fate and transport in shales and tight rocks. Further studies are needed to assess the metal coordination environment as a function of pore size, and correlate any changes in coordination number or bond angles or distances to the observed changes in macroscopic adsorption behavior.

## Additional file

**Additional file 1: Figure S1.** A copper speciation diagram as a function of solution pH. **Figure S2.** The NLDFT pore size distribution plots of SBA-15-8, SBA-15-6, and SBA-15-4. **Figure S3.** A plot showing the Langmuir equilibrium parameter, R_L_, versus the initial copper concentration.
