# Supplementary material for: Adsorption of copper (II) on mesoporous silica: the effect of nano-scale confinement
Source: Geochem Trans. 2018 Jun 26;19:13. doi: 10.1186/s12932-018-0057-4 (PMC6019665; doi:10.1186/s12932-018-0057-4)
Supplement: Supplementary file 1 — Additional file 1: Figure S1. A copper speciation diagram as a function of solution pH. Figure S2. The NLDFT pore size distribution plots of SBA-15-8, SBA-15-6, and SBA-15-4. Figure S3. A plot showing the Langmuir equilibrium parameter, RL, versus the initial copper concentration. [file 12932_2018_57_MOESM1_ESM.docx]

Additional file 1

Adsorption of Copper (II) on Mesoporous Silica: The Effect of Nano-scale Confinement

Andrew W. Knight^1^, Austen B. Tigges^1^, and Anastasia G. Ilgen*^1^

## Cu^2+^ Speciation Diagram

A diagram of Cu^2+^ aqueous speciation as a function of pH was generated with Visual MINTEQ 3.1 using the input concentrations reflecting the experimental set up at pH values ranging from 1 to 14 at [Cu^2+^] = 0.1 mM. This speciation diagram was used to determine the pH for adsorption and kinetics studies, pH = 6 was chosen because Cu^2+^ is the dominant species and adsorption is maximized.


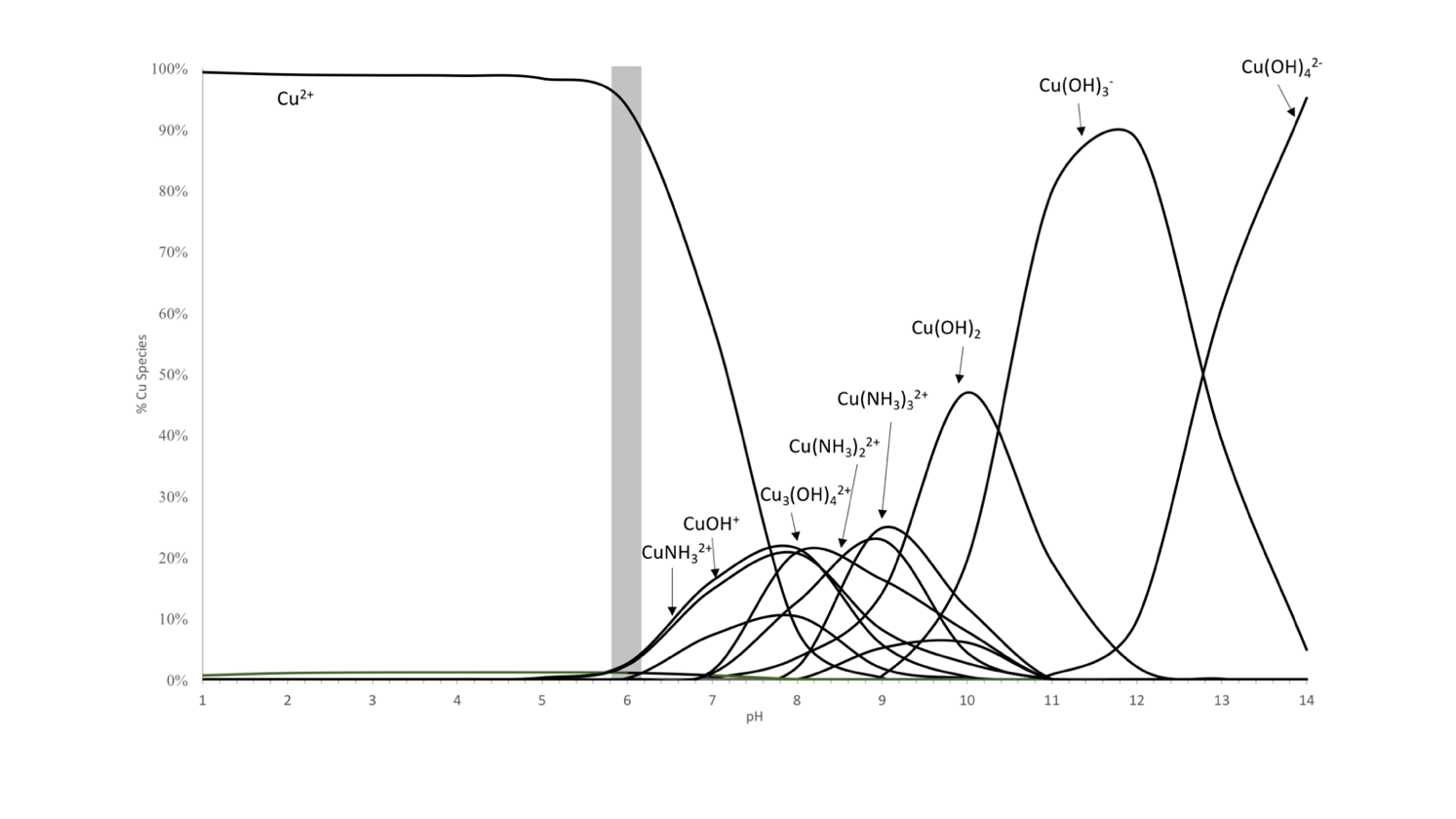


Figure S1. Cu^2+^ Speciation Diagram as a function of pH. The input concentrations were the initial concentrations of all aqueous speciation in the experimental system. Equilibrium speciation was determined with Visual MINTEQ.

## Pore Size Distributions

The pore size distribution of SBA-15-8, SBA-15-6, and SBA-15-4 was determined via NLDFT method by adsorption with N_2_.


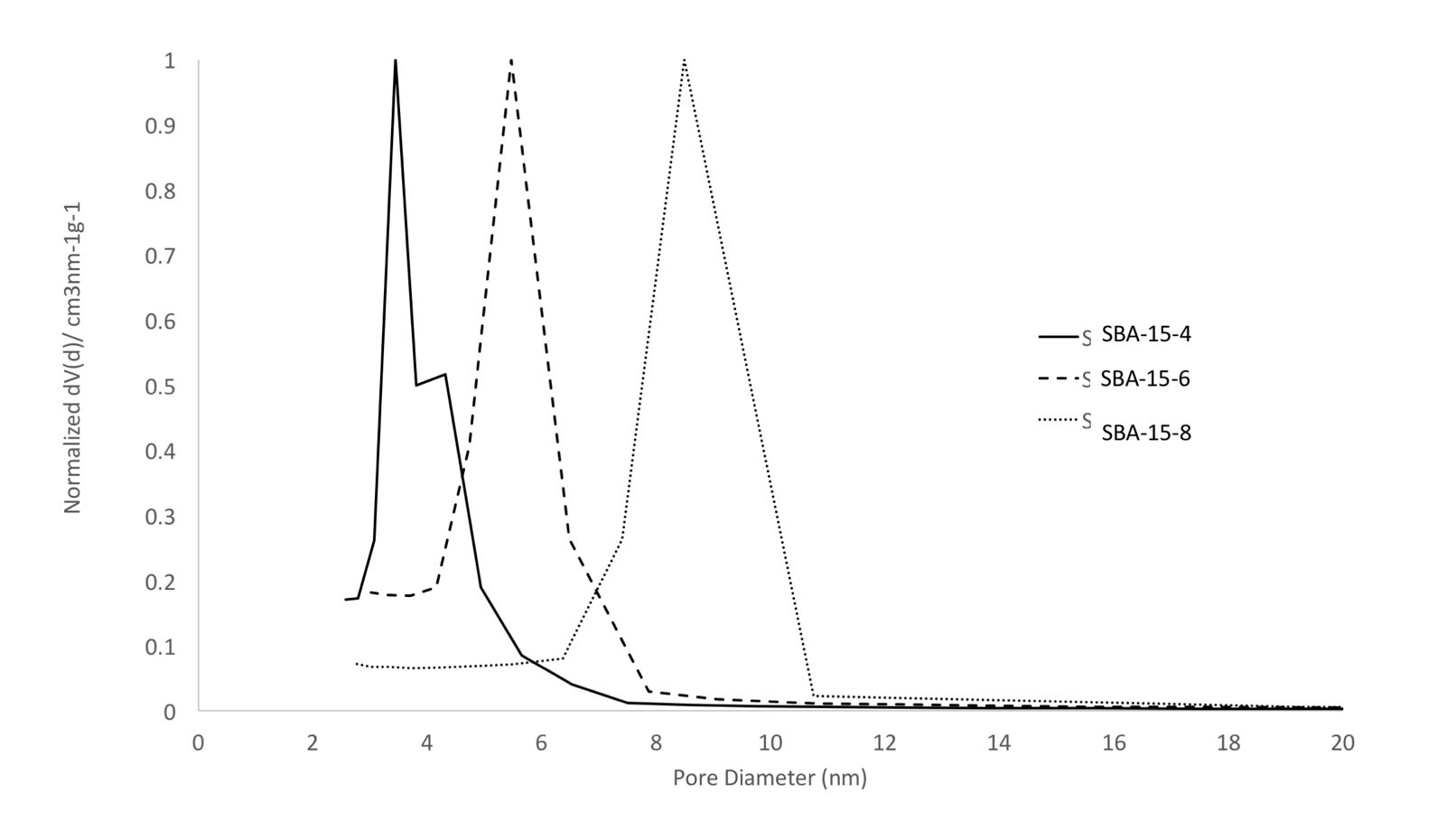


Figure S2. Pore size distribution plot of SBA-15-8, SBA-15-6, and SBA-15-8.

## Adsorption of Cu^2+^ on Mesoporous Materials: Evidence of Nano-scale Confinement

The Langmuir constant, R_L_, was determined for SBA-15-8, SBA-15-6, and SBA-15-4 as a function of the initial [Cu^2+^].


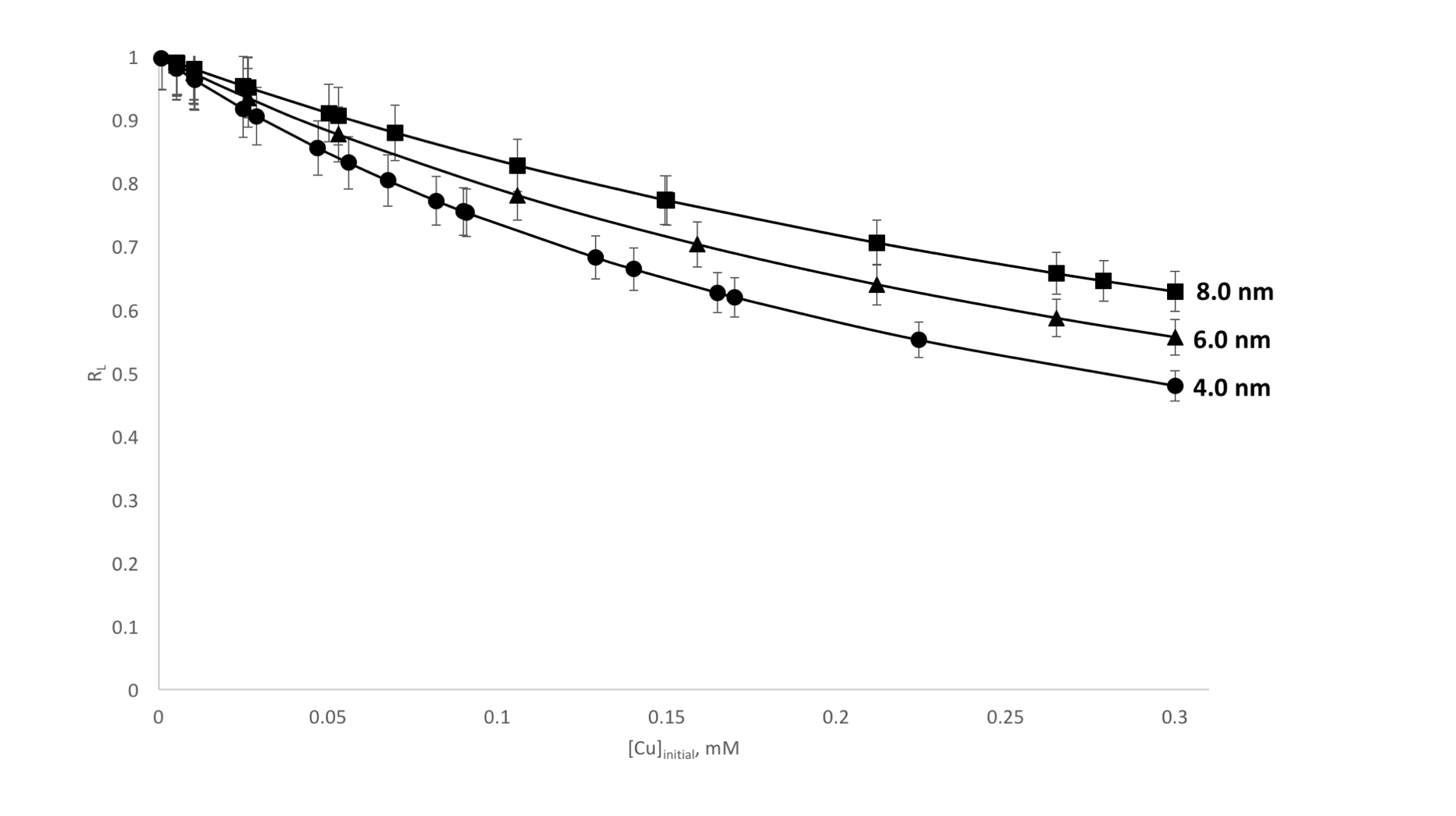


Figure S3. R_L_ values versus [Cu]_initial_ SBA-15-4, SBA-15-6, and SBA-15-8.
